# High-Entropy Alloys and Their Derived Compounds as Electrocatalysts: Understanding, Preparation and Application

**DOI:** 10.3390/ma18174021

**Published:** 2025-08-27

**Authors:** Xianjie Yuan, Xiangdi Yin, Yirui Zhang, Yuanpan Chen

**Affiliations:** 1 School of Mechanical Engineering, North China University of Water Resources and Electric Power, Zhengzhou 450045, China; 17504341325@163.com (X.Y.); c1827892921@163.com (Y.C.); 2 Smart Manufacturing Institute, Zhengzhou University of Economics and Business, 2 Shuanghu Ave, Zhengzhou 451191, China; 18860371233@163.com

**Keywords:** high-entropy alloys, catalyst, rational design, preparation, application

## Abstract

High-entropy alloy (HEA) catalysts have attracted significant attention from researchers. In many cases, HEAs exhibit high activity and selectivity for catalytic reactions due to four “core effects”: high entropy effect, lattice distortion effect, slow diffusion effect, and mixing effect. However, a systematic summary of HEA catalyst design and understanding is lacking. In this review, the reasons for the outstanding performance of HEA catalysts are first discussed from multiple perspectives, such as excellent mechanical properties, ultra-high-performance stability, and the potential for compositional optimization. Furthermore, to deepen our understanding of HEA catalysts, the rational design of HEA catalysts is introduced, covering design principles, element selection, and the use of algorithms for prediction. Next, several common preparation methods for HEAs are introduced, including chemical co-reduction, solution combustion, mechanical alloying, and sol–gel methods. Finally, the research progress of HEA catalysts in hydrogen evolution reactions, oxygen evolution reactions, and oxygen reduction reactions is presented. Unlike existing reviews, this work establishes a unified framework connecting HEA fundamentals (entropy effects), computational design, scalable synthesis, and application-specific performance, while identifying underexplored pathways like lattice-oxygen-mediated mechanisms (LOM) for future research.

## 1. Introduction

In today’s society, with the advancement of technology, energy is an indispensable part of development [1]. However, along with development comes the increasing scarcity of traditional non-renewable energy sources, such as fossil fuels, as well as the greenhouse effect and environmental pollution caused by their excessive use. Therefore, humanity is eager to find alternative green and eco-friendly energy sources to replace traditional non-renewable ones. As a result, electricity, as a clean energy source, has come into human focus and is regarded as the core driving force of the modern industrial system [2]. Specifically, electricity can be produced through diverse and ecologically friendly pathways, such as wind power, hydropower, photovoltaic power generation, and nuclear energy conversion [3]. Notably, the application scenarios of electricity already cover numerous fields including lighting systems, chemical production, textile industry, and information transmission. In the field of clean energy conversion technologies, recent research confirms that electrochemically driven energy can participate in various important reaction systems, including but not limited to: water electrolysis for hydrogen production [4], which utilizes electricity to split water into hydrogen and oxygen, producing green hydrogen (H_2_) as a clean fuel or industrial raw material, achieving zero carbon emissions when combined with renewable energy sources (such as wind and solar) [5]; electrochemical CO_2_ reduction reaction (CO_2_RR) [6], which converts CO_2_ into high-value-added chemicals (such as CO, formic acid, methane, ethylene, etc.), reducing greenhouse gas emissions while synthesizing fuels or chemical feedstocks [7]; electrochemical ammonia synthesis (NRR) [8], which utilizes electricity to convert nitrogen gas (N_2_) and water into ammonia (NH_3_) under ambient temperature and pressure, replacing the traditional high-energy-consumption Haber–Bosch process to achieve green ammonia production (used for fertilizers or hydrogen carriers); and metal–air batteries (e.g., zinc–air batteries), characterized by exceptional energy density (>400 Wh kg^−1^), enabling their deployment in electric transportation systems and large-scale stationary grid storage applications. Catalysts function by lowering reaction energy barriers, enhancing kinetics, and steering reaction pathways, thereby optimizing energy utilization efficiency and product distribution selectivity beyond thermodynamic constraints [9]. The following presents the application prospects of electrocatalysis as shown in Table 1.

Currently, many researchers are engaged in designing electrocatalysts with excellent activity, low cost, and high stability [13,14,15]. Common electrocatalysts include precious metal-based catalysts [16,17], transition metal-based catalysts [18], carbon-based catalysts [19], MOFs/COFs [20], single-atom catalysts [21], and non-metallic catalysts, each with distinct advantages and disadvantages [22], as listed in Table 2, which compares the pros, cons, and application scenarios of these six catalyst types.

**Figure 1:**
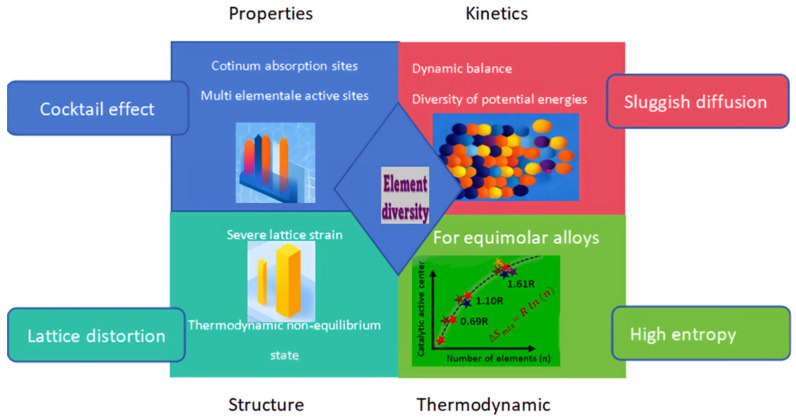
The core effects of high-entropy alloys (HEAs).

Pioneering research by Yeh et al. in 2004 established the foundational paradigm of high-entropy alloys [23]. This materials innovation transcends conventional alloy design frameworks through configurational entropy stabilization, enabling unprecedented compositional space exploration. Their defining feature resides in single-phase solid solutions stabilized by configurational entropy, comprising ≥ 5 principal constituents with equimolar/near-equimolar stoichiometry (each element atomic percentage between 5 and 35%) [24]. Unlike traditional alloys designed with 1–2 primary elements, HEAs achieve uniform mixing of multiple elements and highly disordered atomic arrangements. These alloys predominantly form entropy-stabilized single-phase structures with high-symmetry crystallographic motifs (FCC, BCC, or HCP), effectively suppressing the formation of intricate intermetallic compounds and amorphous phases through entropic selection. This characteristic significantly enhances the comprehensive properties of the material [25]. Furthermore, HEAs have become a research hotspot in materials science due to their unique “four core effects” [26]. High-entropy alloy catalysts exhibit ultra-high activity and stability in many reactions, including hydrogen evolution reaction [27,28], oxygen evolution reaction [29], oxygen reduction reaction [30], carbon dioxide reduction reaction, nitrogen reduction reaction [31,32], methanol oxidation reaction [33], ethanol oxidation reaction [34], glycerol oxidation reaction [35], etc. To understand the underlying principles of these reactions, explore why high-entropy alloys can serve as excellent catalysts, and comprehend how to fabricate or design stable, high-performance HEA catalysts, this paper provides a comprehensive review based on recent reports and publications on the understanding, fabrication, and application of HEAs as superior catalysts. While prior reviews focus on HEA synthesis or specific applications (e.g., HER or OER), a systematic analysis linking the ‘four core effects’ of HEAs to electrocatalytic mechanisms, rational design algorithms (e.g., ML/DFT), and industrial-scalability challenges of preparation methods is lacking. The following Figure 1 intuitively introduces the core role of high-entropy alloys.

## 2. Fundamental Principles of HEA Catalysts

### 2.1. Superior Catalytic Performance of HEAs

In the field of catalysis research, researchers have long been dedicated to developing high-activity and high-stability catalysts. Due to the “four core effects” and their recognition as excellent heterogeneous catalyst candidates, this section will explain the reasons for the superior catalytic performance of HEAs.

#### 2.1.1. Outstanding Mechanical Properties

HEA catalysts exhibit exceptional mechanical properties owing to their unique multi-element synergistic effects and high-entropy effect. For instance, multi-element alloys like FeCoNiCrMn, after surface modification, can form nanostructures with high-density active sites, significantly enhancing their catalytic efficiency in the oxygen evolution reaction and hydrogen evolution reaction, with overpotentials as low as 22 mV (in 0.5 M H_2_SO_4_ at 25 °C) [36]. The multi-component nature endows HEAs with high strength and ductility during deformation. For example, the CoCrFeNi alloy maintains good toughness even at low temperatures, with a fracture toughness exceeding 200 MPa·m^1/2^, far higher than traditional alloys [37]. In the catalytic field, the actual performance and effectiveness of catalysts are inseparable from their mechanical strength parameters. These parameters, such as compressive strength (typically requiring 80–150 N/cm^2^ or ≮120 N/particle standards), attrition rate, and particle shape and size (attrition rate needs to meet industry standards, generally ≤3.0%; bulk fracture needs extra attention in high-temperature fluidized environments; particle shape preferably ring-shaped or trefoil for low pressure drop and high specific surface area; particle size distribution width (PDI (particle dispersion index) < 0.2) should be strictly controlled, with fixed beds preferring 3–8 mm and fluidized beds focusing on 50–100 μm), are key indicators for measuring catalytic activity. In catalyst systems, practical performance is closely related to mechanical strength parameters, which serve as key indicators of catalytic activity. Understanding these parameters enables analysis of catalyst behavior and operating conditions, making them essential for optimized catalyst design and application.

#### 2.1.2. Ultra-High-Performance Stability

Equally important, HEAs typically possess ultra-high stability in high-temperature and corrosive media, primarily attributed to their sluggish diffusion effect. For example, adding elements like Nb and Ti, as in the FeCoCrNiMnNb_2_ alloy, can further refine grains and accelerate the formation of passivation films, thereby enhancing corrosion resistance [38]. In extreme corrosive environments, such as high-temperature salt solutions or acidic media, HEAs maintain stable corrosion resistance in extreme environments due to high configurational entropy and “cocktail effect”, making the material less prone to micro-galvanic corrosion in complex environments. In catalysis, HEA nanocatalysts form a bilayer nanostructure on the surface, not only improving the catalytic activity for OER but also possessing a dynamic compensation mechanism ensuring long-term stability. In terms of mechanical properties, HEAs combine high strength, high hardness, and good ductility. For instance, the CoCrFeNi alloy maintains excellent creep resistance at high temperatures, with significantly higher yield strength and ultimate strength than traditional alloys [39]. This ultra-high stability enables high-entropy alloys to be applied in harsh catalytic reactions under real-world conditions.

#### 2.1.3. Optimizable Composition and Structure

Traditional binary and ternary alloys have limited continuous adjustability of their composition. In contrast, HEAs with four or more elements allow for continuous tuning and modification of their composition and structure. Through these flexible adjustments, the catalytic activity of HEAs can be finely tuned with ease. The vast compositional space of multi-component HEAs provides numerous possibilities for designing HEA catalysts.

From a compositional perspective, the precise regulation of the d-band center through multi-element synergy is the core mechanism for optimizing the electronic structure. The position of the d-band center of transition metals directly affects the adsorption strength of reaction intermediates: an upward shift enhances adsorption energy, while a downward shift weakens it [40]. HEAs achieve continuous tunability of the d-band center through electron transfer between multi-component elements (e.g., high work function elements Pt/Ni with low work function elements Fe/Co/Cu). For example: Tian et al. demonstrated through a series of HEA catalysts (FeCoNiMn > PtIr > FeCoNiMnPtIr, etc.) that the d-band center exhibits a volcano-type relationship with catalytic activity in lithium-oxygen batteries (LOB); a moderate d-band center optimizes LiO_2_ adsorption energy, yielding the lowest overpotential [41]. Density functional theory calculations further revealed that the d-band center of Au_0.5_Ir_0.5_HEA shifts down by 0.5 eV compared to pure Pt, reducing the adsorption energy of oxygen intermediates by 0.2 eV, enhancing oxygen reduction reaction activity. In ammonia synthesis, the Pt_0.8_Fe_0.2_Co_0.2_Ni_0.2_Cu_0.2_ intermetallic compound, with a d-band center at −2.24 eV, lowers the dissociation energy of the N-H bond, resulting in an energy barrier of only 0.23 eV for the NO→NOH step, far superior to single-metal catalysts [42]. Machine learning-assisted multi-objective optimization can efficiently screen d-band center positions, predicting adsorption enthalpies with errors <0.16 eV, highlighting the trend towards intelligent compositional design [43].

From a structural perspective, the diversity and synergistic effects of active sites are key factors. HEAs composed of five or more principal elements exhibit high surface heterogeneity, forming a large number of active sites with different local coordination environments [44]. This structural heterogeneity allows reactant molecules (e.g., H_2_O, O_2_, N_2_) to achieve differentiated adsorption and activation at different sites, breaking the limitations of linear adsorption energy relationships in traditional catalysts. For example: Feng et al. confirmed via in situ X-ray absorption spectroscopy (XAS) that in FeCoNiRhPt ultra-small HEA nanoparticles, Pt and Rh sites dominate hydrogen adsorption dissociation and the overall reaction path, respectively, while Fe/Co/Ni sites indirectly enhance activity by modulating the electronic structure of neighboring sites [45]. Furthermore, the selection of constituent elements and their molar ratios critically governs the population of active sites and their local atomic coordination, consequently modulating the intrinsic activity of these sites [46]. For example: Zhan et al. developed subnanometer nanowires (SNWs) composed of PtRuNiCoFeMo high-entropy alloy (HEA) for the alkaline hydrogen oxidation reaction [47]. The resulting HEA SNWs/C catalyst exhibited mass and specific activities reaching 6.75 A mg^−1^ and 8.96 mA cm^−2^, respectively, which represent mass activity enhancements of 2.8-fold, 4.1-fold, and 19.8-fold, and specific activity improvements of 2.6-fold, 2.4-fold, and 18.7-fold relative to HEA NPs/C, commercial PtRu/C, and Pt/C, respectively. Furthermore, nanoporous structure design can increase the specific surface area up to 70 times that of traditional alloys, and surface roughness promotes reactant diffusion, significantly increasing the exposure density of active sites [48]. This multi-site synergistic mechanism is particularly prominent in electrocatalytic water splitting. For instance, the CoCrFeNiNb HEA operates stably for over 100 h at a current density of 100 mA cm^−2^, attributed to the dynamic complementary effect of active sites.

Notably, significant functional differentiation exists among sites in catalytic systems: some sites serve as direct active centers dominating the reaction process, while others participate in system regulation but are not direct catalytic sites. Such non-direct active sites may indirectly influence reaction efficiency by modulating electronic structure, adsorption behavior, or spatial conformation. For example: Feng et al. found in NiCoFePtRh HEA-NPs for HER, through in situ characterization, that Pt and Rh are prominent active sites [45].

### 2.2. Rational Design of HEA Catalysts

The rational design of HEA electrocatalysts remains a challenge. Designing HEA electrocatalysts first requires satisfying the compositional conditions for HEAs: (1) Atomic radius difference (δ) ≤ 6.6%(2) −11.6 < Mixing enthalpy (ΔH) < 3.2 kJ mol^−1^. In current research, most HEA electrocatalysts are primarily composed of precious metals (e.g., Pt, Pd, Ru, Rh, Au, Ag, Ir) combined with abundant transition metals including Mg, Al, Ni, Co, Cu, Cr, Fe, and Mn [45]. In catalyst design, combining precious metals with transition metals often reduces material costs and enhances catalytic activity through synergistic effects. For HEA electrocatalyst design, priority should be given to selecting elements with similar atomic radii and potential regulatory effects on the target reaction. The relative content of these elements should be precisely controlled to optimize the adsorption energy of key intermediates, thereby improving overall catalytic performance.

Advanced computational approaches—including Density Functional Theory (DFT), Molecular Dynamics (MD), Monte Carlo techniques, High-Throughput Screening (HTS), Machine Learning (ML), and Artificial Neural Networks (ANNs)—enable insights into catalytic mechanisms and rational catalyst design [49]. These methodologies enable both accelerated identification of optimal constituent elements for HEAs and quantitative determination of adsorption energetics for critical intermediates on catalytic surfaces, which subsequently facilitates pathway elucidation and mechanistic deciphering [50,51]. Among them: DFT is based on quantum mechanics principles, predicting material ground-state properties (e.g., band structure, elastic modulus, phase stability) by calculating electron density distributions, providing atomic-scale mechanistic explanations for hard coating design (e.g., transition metal nitrides) [52]. MD simulation numerically solves Newton’s equations of motion, simulating the trajectories of atoms/molecules under classical force fields or quantum potential energy surfaces, revealing material microscopic deformation mechanisms (e.g., dislocation evolution in Ni-based superalloys, fracture behavior of graphene) and thermodynamic properties [53]. Monte Carlo Simulation uses random sampling to build probability models, simulating material microstructure evolution (e.g., precipitate nucleation in aluminum alloys), cumulative error analysis in tolerance design, and uncertainty quantification in mechanical system reliability assessment [54]. HTS combines automated experimental/computational platforms (e.g., combinatorial chemical deposition, CALPHAD phase diagram calculation) to test thousands of material formulations in parallel, rapidly identifying components with target properties (e.g., catalyst mechanical strength, cartilage tissue engineering materials), significantly shortening R&D cycles [55]. ML utilizes data-driven models (e.g., support vector machines, decision trees) to mine structure-property relationships between material microscopic features and macroscopic performance, enabling rapid prediction of properties like elastic modulus and strength based on high-throughput databases, replacing traditional trial-and-error experiments [56]. ANNs learn nonlinear mapping relationships through multi-layer perceptron structures, predicting mechanical properties of steel alloys (e.g., yield strength, elongation) based on chemical composition/heat treatment parameters, or directly outputting the effective stiffness of composites based on microstructure images.

For example, Pedersen et al. utilized first-principles-based density functional theory (DFT) to compare HEAs with pristine Cu(111) surfaces, establishing the reasons behind the catalytic activity and selectivity of HEAs for CO_2_ electroreduction [57]. They combined DFT with machine learning to study CO and CO_2_ reduction reactions on HEAs. Ultimately, they demonstrated that nanocrystalline equiatomic Au-Ag-Pt-Pd-Cu HEAs prepared by a low-temperature method exhibit unprecedented catalytic activity for efficient electrochemical CO_2_RR. Based on free energy calculations of intermediates, DFT studies showed that HEAs are superior catalysts compared to pristine Cu metal. By optimizing the composition of the HEA, the side effects of HEAs were suppressed, and the selectivity for CO and CO_2_ reduction was improved.

**Figure 2:**
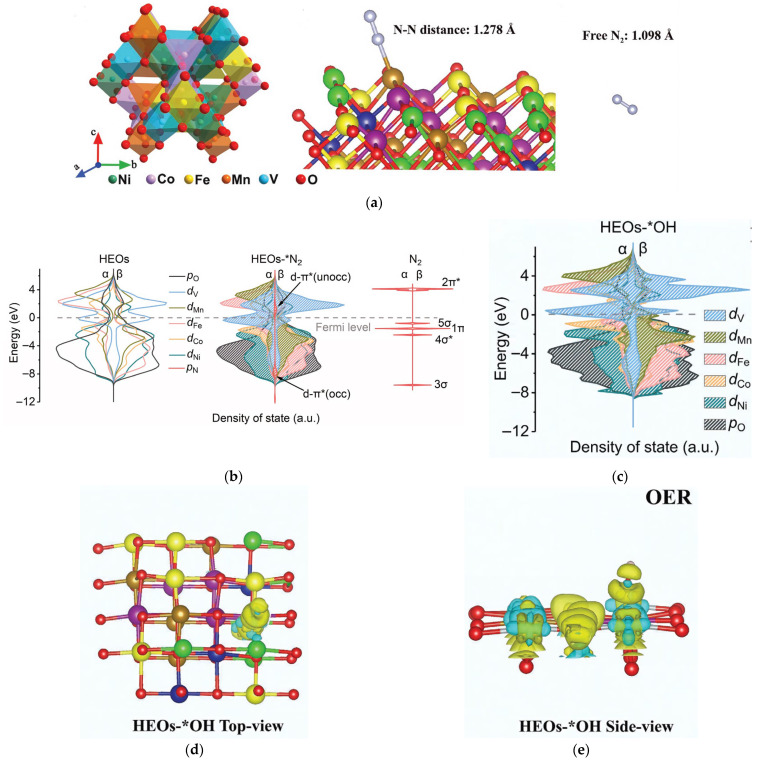
(**a**) Crystal structure of spinel HEO and model of *N_2_ and free N_2_. (**b**) Simulated DOS near the Fermi level of HEO before and after N_2_ adsorption. (**c**–**e**) Prediction of anodic OER activity: (**c**) Simulated DOS near the Fermi level of HEOs after adsorbing OH^−^. Charge density difference map of *OH in (**d**) top view (**e**) side view. Developed on the basis of, and reprinted with permission from, Ref. [31]. Copyright © 2022 Wiley-VCH GmbH.

To engineer bifunctional catalysts exhibiting superior oxygen evolution reaction (OER) and nitrogen reduction reaction (NRR) activity, Sun et al. employed density functional theory (DFT) calculations to identify high-entropy oxides (HEOs) incorporating Ni-Fe-Co-Mn-V constituents as promising candidates for dual-functional catalysis. Subsequent experimental synthesis was conducted to validate this computational prediction [31]. They established a theoretical model of single-phase spinel-structured HEOs composed of 1/3 M(II)O and 2/3 M(III)O. Density of States (DOS) and differential charge density were used to predict the N_2_ activation capability of the HEO surface, as shown in Figure 2a. Subsequently, the DOS analysis revealed dominant contributions from Ni/Co/Fe/Mn/V d-orbitals and O p-orbitals near the Fermi level in these HEOs. This d-p orbital hybridization creates highly delocalized electronic states, endowing the catalysts with enhanced electron mobility critical for facilitating nitrogen activation during NRR processes. The DOS spectra after N adsorption onto the HEO surface were analyzed, DFT analysis reveals significant spin polarization in vanadium d-orbitals near the Fermi level following N-adsorption, with distinct α/β orbital splitting facilitating N≡N bond elongation through spin-selective electron donation. This electronic reorganization enhances d-π* backdonation, directly weakening the triple bond (Figure 2b). Complementary charge density difference maps (Figure 2c–e) demonstrate pronounced charge accumulation at oxygen sites and depletion from transition metal centers, confirming ligand-to-metal charge transfer during activation. The vacant d-orbitals of transition metals serve as electron acceptors for oxygen-derived electron density, endowing these sites with enhanced proton-coupled electron transfer capability that facilitates adsorbed *OH deprotonation during initial OER steps. Ultimately, they successfully confirmed the initial prediction and synthesized HEOs in the form of sea urchin-like hollow nanospheres assembled from 2D nanosheets. These HEOs exhibited excellent NRR, OER, and neutral water electrolysis catalytic activity.

## 3. Preparation Methods of High-Entropy Alloys

Recently, an increasing number of researchers have focused on HEA catalysts, and HEAs have also excelled in various fields of catalysis. This has led to the exploration of various preparation methods, such as chemical co-reduction, solution combustion, mechanical alloying, vacuum arc melting, and sol–gel techniques. Due to the countless element combinations, researchers have various methods to tune the electronic state and structure of HEAs. Owing to the four “core effects” of HEAs, compared to single metals and phase-separated alloys, HEA catalysts exhibit superior activity and stability in various electrocatalytic reactions. The preparation methods of HEAs are discussed below.

### 3.1. Chemical Co-Reduction Method

Chemical co-reduction is a method for preparing high-entropy alloy (HEA) catalysts by simultaneously reducing multiple metal precursors via solvothermal or wet-chemical strategies [58]. Its core lies in utilizing the synergistic effect of multi-metal systems and the high-entropy effect (i.e., high configurational entropy suppressing phase separation) to achieve co-reduction and uniform mixing of different metal elements in solution, forming single-phase or multi-phase solid solution structures [59]. This method requires precise control of metal salt precursors, reducing agents, chelating agents, and reaction conditions to overcome kinetic imbalances caused by differences in the intrinsic reduction potentials of different metals. Local non-equilibrium environments (e.g., active hydrogen generation) promote synchronous co-reduction and inhibit phase separation.

The advantages of the chemical co-reduction method for preparing HEA catalysts are mainly reflected in its compositional flexibility and structural homogeneity [60]: By precisely adjusting precursor ratios or introducing chelating agents (e.g., ethylenediaminetetraacetic acid, EDTA), precise atomic-scale control of multi-metal element ratios can be achieved. Simultaneously, non-equilibrium reduction environments (e.g., active hydrogen generation or laser-induced conditions) suppress thermodynamic phase separation, promote uniform element distribution, and form stable solid solution structures with high configurational entropy. Additionally, lattice distortion and electronic structure optimization induced by multi-element synergistic effects can significantly enhance catalytic activity and stability. For example, the sluggish diffusion effect of HEAs can delay catalyst surface deactivation. However, this method still faces dual challenges of reduction kinetic imbalance and process complexity: Differences in intrinsic reduction potentials lead to preferential reduction of some metals, requiring reliance on chelating agents or special reaction conditions (e.g., local high temperature/pressure) to control the reduction path and maintain synchronous co-reduction [61]. Simultaneously, the synthesis process requires strict control of parameters such as precursor concentration, temperature gradients, and reducing agent concentration. For instance, the fine control of surfactants and dealloying conditions in oil-phase co-reduction increases process difficulty. Moreover, techniques like solvothermal methods have high equipment requirements, making homogeneity control and large-scale production stability bottlenecks that limit their practical application range.

**Figure 3:**
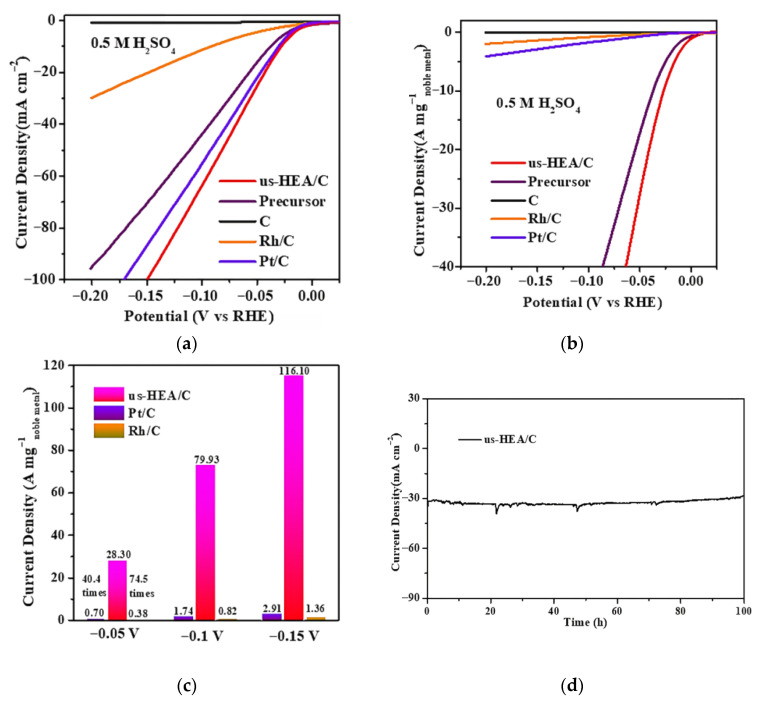
All HER tests performed in 0.5 M H_2_SO_4_ at 25 °C. (**a**,**b**) HER polarization curves and ECSA-normalized HER polarization curves of NiCoFePtRh HEA nanoparticles and their counterparts. (**c**) ECSA-normalized activity comparison at different potentials. (**d**) HER stability test of NiCoFePtRh HEA nanoparticles. Reprinted with permission from Ref. [45]. Copyright © 2021, American Chemical Society.

Feng et al. employed this versatile and facile chemical co-reduction method to prepare a series of carbon-supported ultra-small high-entropy alloy (us-HEA) nanoparticles (NPs) [45]. The carbon-supported high-entropy alloy nanoparticulates (NiCoFePtRh/C) demonstrated exceptional dispersion homogeneity, exhibiting a record-setting average diameter of 1.68 nm—the minimal nanoscale architecture documented in HEA systems to date. HAADF-STEM and XAFS measurements revealed the atomic structure, electronic structure, and coordination structure of the us-HEAs. The us-HEA/C catalyst delivered exceptional mass activity (28.3 A mg^−1^ at −0.05 V vs. RHE), surpassing commercial Pt/C and Rh/C by factors of 40.4× and 74.5×, respectively (Figure 3a–c). At η = 50 mV, it achieved a turnover frequency (TOF) of 30.1 s^−1^, representing a 41.8-fold enhancement over Pt/C. Remarkably, the catalyst retained >99% initial activity after 10,000 potential cycles (Figure 3d), demonstrating unparalleled durability. In situ X-ray absorption spectroscopy and theoretical calculations revealed the actual active sites, fine-tuning of the electronic structure, and synergistic effects among the five elements, which endowed the us-HEA with significantly enhanced HER activity. The exceptional performance of these supported us-HEA NPs makes them potentially applicable as advanced HER catalysts for future renewable energy storage.

**Figure 4:**
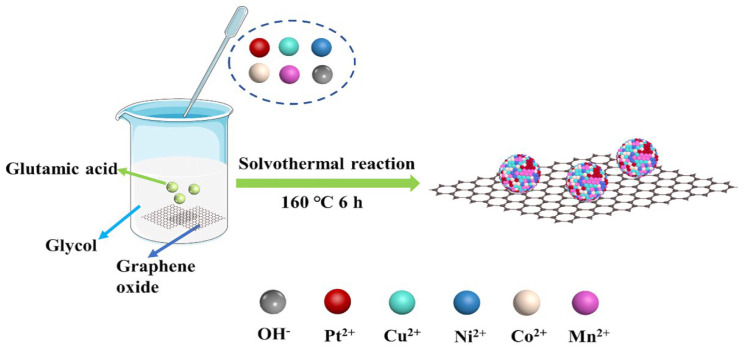
Schematic diagram of the synthesis of PtCuNiCoMn HEA nanoparticles on rGO. Reprinted with permission from Ref. [62]. Copyright © 2024, American Chemical Society.

Cheng et al. synthesized PtCuNiCoMn HEA nanoparticles supported on reduced graphene oxide (rGO) via co-reduction and used them as electrocatalysts for methanol and formic acid electrooxidation (Figure 4) [62]. The PtCuNiCoMn/rGO catalyst exhibited a specific activity of 789.4 mA mg^−1^, higher than PtCuNiCo/rGO, PtCuNi/rGO, PtCu/rGO, Pt/rGO, and commercial Pt/C catalysts. It also demonstrated better resistance to CO poisoning. Furthermore, chronoamperometry and cyclic voltammetry tests revealed the better stability of the PtCuNiCoMn/rGO catalyst. XPS results indicated that the electronic structure of Pt in PtCuNiCoMn HEA nanoparticles changed due to synergistic effects among the constituent metals, thereby enhancing the reaction kinetics of methanol and formic acid electrooxidation on Pt. Moreover, due to the high-entropy effect and sluggish diffusion effect, PtCuNiCoMn HEA nanoparticles exhibited good compositional stability during electrochemical testing.

**Figure 5:**
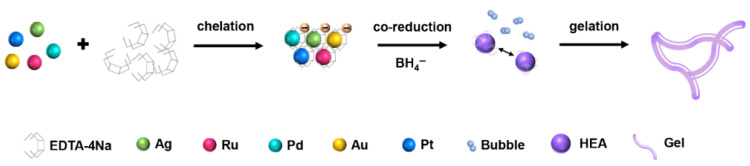
Principle of AgRuPdAuPt aerogel formation. Reprinted with permission from Ref. [63]. Copyright © 2023, American Chemical Society.

Nie et al. synthesized a high-entropy alloy aerogel, AgRuPdAuPt, via co-reduction (Figure 5) [63]. The optimized AgRuPdAuPt exhibited ultra-small overpotentials of 22 mV (at 10 mA cm^−2^) and excellent stability for 24 h in 1.0 M KOH electrolyte.

**Figure 6:**
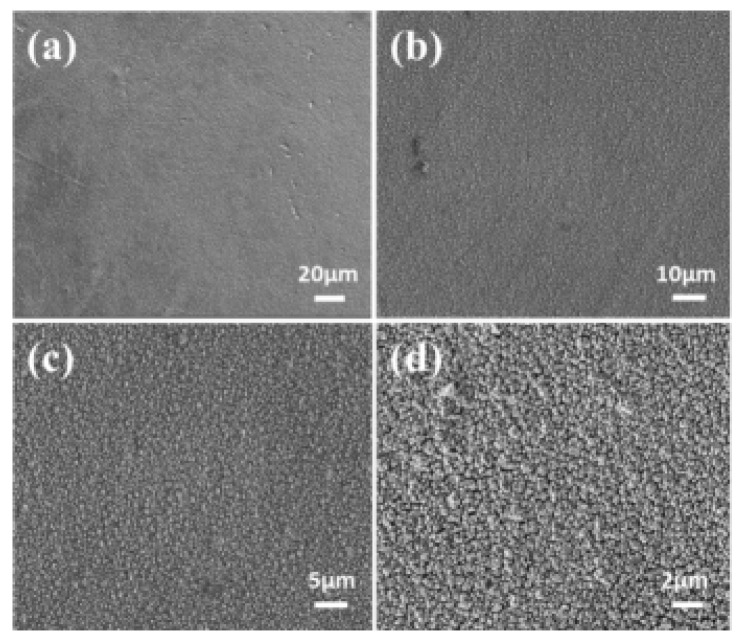
SEM morphological images of Fe-Co-Ni-Cu-Zn high entropy alloy films at different magnifications., (**a**) 20 microns. (**b**) 10 microns. (**c**) 5 microns. (**d**) 2 microns. Reprinted with permission from Ref. [61] Open access.

Zhou et al. prepared nanoscale Fe-Co-Ni-Cu-Zn HEA films via potentiostatic electrochemical deposition in an aqueous electrolyte (Figure 6) [61]. The films consisted of particles with sizes ranging from approximately 200 to 550 nm and formed a single face-centered cubic (FCC) solid solution phase structure. They exhibited significant soft magnetic properties (saturation magnetization of 23.22 emu/g, coercivity of 90 Oe) and good electrical performance (resistivity of 4.67 mΩ∙cm).

### 3.2. Solution Combustion Synthesis

Solution combustion synthesis (SCS) for preparing HEA catalysts is an efficient synthesis strategy based on the rapid self-propagating combustion reaction of metal nitrates with organic fuels (e.g., glycine) [64]. It enables precise control of pore structure, specific surface area, and element distribution by adjusting the molar ratio of metal salts to fuel and the subsequent reduction temperature. For example, the FeCoNiCu_0.5_ HEA, after parameter optimization, exhibits a rich porous structure and high specific surface area (up to 50 m^2^/g), significantly enhancing hydrogen evolution reaction (HER) catalytic activity and stability [65]. Additionally, this method combined with hydrogen reduction processes achieves uniform dispersion of multi-metal components and synergistic effects of surface active sites. DFT calculations show that electronic interactions between Fe, Co, Ni, and Cu elements optimize H* adsorption energy and accelerate H_2_ desorption kinetics [65,66]. However, its disadvantages include high sensitivity to precursor composition ratios and combustion conditions. Imbalance in the fuel-to-metal salt ratio can lead to incomplete combustion or residual carbon impurities, requiring subsequent high-temperature reduction or acid washing to optimize surface cleanliness, increasing process complexity. Moreover, SCS typically relies on rapid exothermic reactions, which may cause element segregation or grain coarsening due to local temperature gradients. Dynamic quenching techniques need to be combined to inhibit grain growth and maintain nanostructures [66,67].

The preparation of precursors is crucial as it directly determines the phase purity, microstructure, and performance of the final product. Precursors, as organic polymers or salt mixtures containing target metal elements (e.g., composite systems of metal nitrates and fuels), benefit from molecular-level homogeneous mixing, significantly shortening diffusion distances between elements. This reduces the temperature required for subsequent combustion reactions (typically from over 1000 °C in traditional methods to 350–500 °C), effectively inhibiting abnormal grain growth and ensuring uniform phase composition and fine grain size [68]. For example, in preparing ceramic powders, the distribution uniformity of metal ions in the precursor directly affects the specific surface area and pore structure of the powder after combustion-co-precipitation for precursors may form larger grains, while the sol–gel method can generate nanostructures with high specific surface area [69]. Furthermore, the chemical state of the precursor (e.g., oxidation degree) plays a key regulatory role in the reaction path: Oxidized precursors with metal content deviating from the theoretical value, if subjected to the calcination regime of unoxidized precursors, will lead to a capacity reduction of about 10 mAh/g. In contrast, high-valence precursors (e.g., Ni_1/3_Co_1/3_Mn_1/3_OOH) compared to low-valence systems (e.g., hydroxides), under the same synthesis conditions, can increase the first discharge capacity of lithium battery cathode materials to 153 mAh/g and improve cycle stability by over 25% [70]. The morphology characteristics of the precursor also influence the final product through a “genetic effect.” Flaky precursors form large-sized grains after calcination, while fine particle precursors generate submicron crystals, with significant differences in compaction density and rate performance. In reaction control, precursor solution concentration, pH, and mixing temperature (e.g., gelation at 80 °C or sol formation at 65 °C) precisely regulate combustion rate and exothermic intensity, avoiding composition segregation caused by local overheating [71]. For instance, preparing aluminum precursors above 95 °C accelerates precipitation but requires weighing yield loss [72]. Therefore, precursor preparation is the core hub balancing reaction kinetics, thermodynamics, and product performance. Its design needs to simultaneously optimize molecular mixing degree, redox characteristics, and morphology control to meet stringent material structure requirements for specific applications.

Common chemicals used for preparing precursors can be categorized as follows: Metal Salts, Fuel, Additives, and Solvent. Metal Salt Solutions: Typically metal nitrates, chlorides, or acetates. Nitrates are preferred due to good water solubility, low decomposition temperature (200–400 °C), and decomposition products (NO_2_, O_2_) promoting combustion. Fuel: Typically urea (CO(NH_2_)_2_), glycine (C_2_H_5_NO_2_), hydrazines (N_2_H_4_), citric acid (C_6_H_8_O_7_), or sucrose/glucose. Solvent: Generally water or organic solvents. Water ensures thorough dissolution of metal salts and fuel; organic solvents enhance fuel solubility. Additives: Include combustion promoters, chelating agents, dispersants, etc., serving various functions.

**Figure 7:**
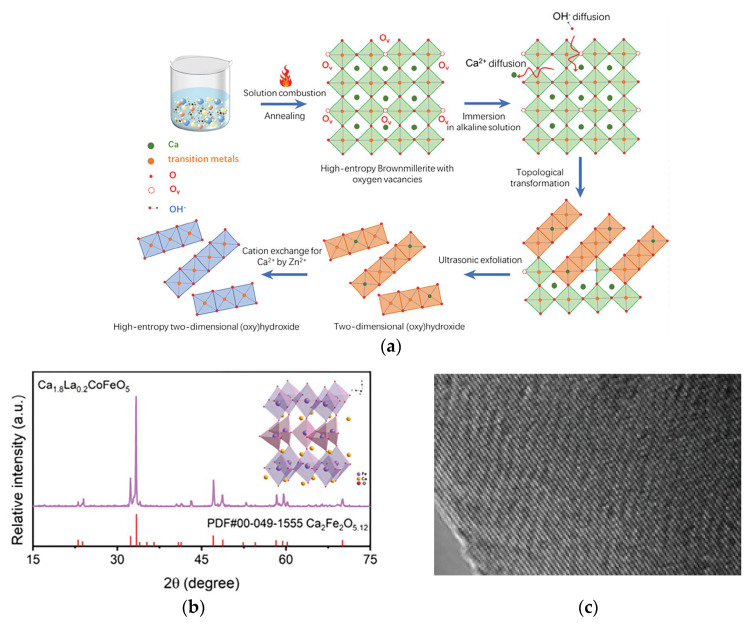

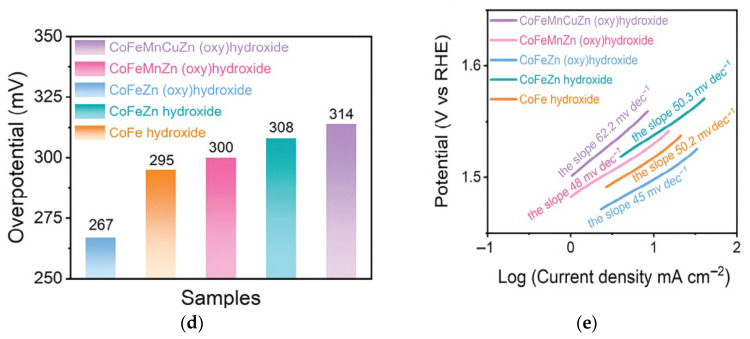
(**a**) Schematic illustrating the synthesis strategy for deriving multi-component transition metal (oxy)hydroxides from brownmillerite oxides. (**b**) XRD patterns, (**c**) TEM image. OER performances were measured in 1 m KOH electrolyte. (**d**) η10, (**e**) corresponding Tafel plots. Reprinted with permission from Ref. [73]. Copyright © 2024 Wiley-VCH GmbH.

Liu et al. prepared Ca-rich brownmillerite oxides via solution combustion synthesis and calcination [73]. The synthesis process for multi-element metal (oxy)hydroxide ultrathin nanosheets is illustrated in Figure 7a. Its flexible structure facilitates easy elemental adjustment. Subsequently, immersing the brownmillerite in alkali induced OH^−^ ion diffusion into the structure and dissolved Ca ions, causing the collapse of the brownmillerite structure and its topological transformation into an (oxy) hydroxide phase. This transformation involved a shift from the original corner-sharing structure of metal-oxygen octahedra to an edge-sharing structure. Using brownmillerite as a precursor, they successfully synthesized CoFeZn ternary, quaternary CoFeMnZn, and pentanary high-entropy CoFeCuMnZn (oxy) hydroxide ultrathin nanosheets with uniform mixing of metal elements. Introducing Zn into CoFe (oxy) hydroxide caused the O 2p band center to shift towards the Fermi level, promoting the lattice oxidation mechanism (LOM) pathway. Direct O-O coupling in LOM was identified, verified by tetramethylammonium cations (TMA^+^) chemical probes detecting peroxide-like (O_2_^2−^) species. Consequently, the catalyst provided an ultra-low overpotential of 267 mV (Figure 7d) and an exceptionally low Tafel slope of 45 mV dec^−1^ (Figure 7e). Due to enhanced resistance to Fe leaching, significant stability was achieved even after 10,000 cyclic voltammetry (CV) cycles, with negligible overpotential difference.

**Figure 8:**
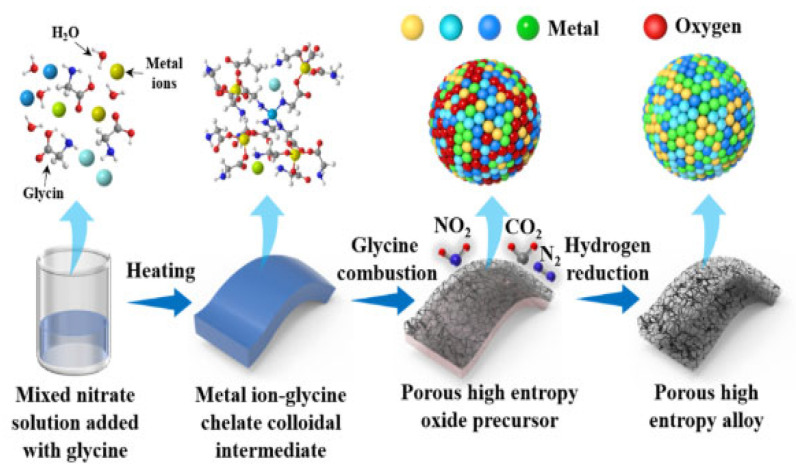
Schematic of the preparation process for porous FeCoNiCu high-entropy alloy. Reprinted with permission from Ref. [65]. Copyright © 2024 Elsevier B.V. All rights are reserved, including those for text and data mining, AI training, and similar technologies.

**Figure 9:**
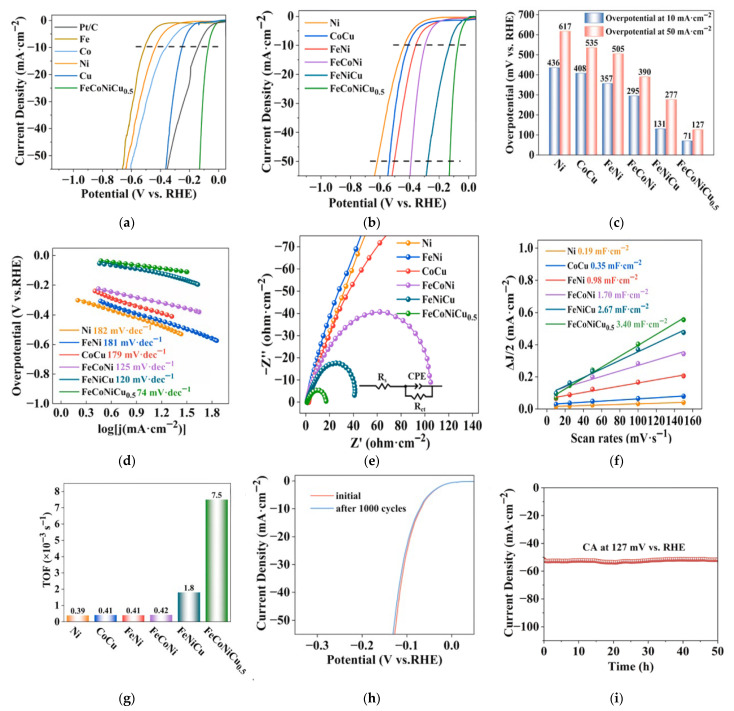
In 1 M KOH, 25 °C (**a**) HER polarization curves of FeCoNiCu and unitary metal catalysts (commercial Pt/C, Fe, Co, Ni, Cu). (**b**) HER polarization curves, (**c**) Tafel plots, (**d**) Local Tafel plot with related linear fitting results, (**e**) Nyquist plots, (**f**) C~dl~ values and (**g**) TOF values at 100 mA cm^−2^ current density for FeCoNiCu and unitary (Ni), binary (FeNi, CoCu), and ternary (FeCoNi, FeNiCu) catalysts. (**h**) HER polarization curves of porous FeCoNiCu HEA before and after 1000 CV cycles. (**i**) Long-term stability test of porous FeCoNiCu HEA at 127 mV vs. RHE. Reprinted with permission from Ref. [65]. Copyright © 2024 Elsevier B.V. All rights are reserved, including those for text and data mining, AI training, and similar technologies.

Guo et al. combined solution combustion and hydrogen reduction methods to prepare porous homogeneous FeCoNiCu high-entropy alloys (Figure 8) [65]. First, composite oxide precursors with tunable elemental molar ratios and porous structures were synthesized using SCS, achieving uniform element distribution through precise control of combustion parameters. Subsequently, the oxide precursors were subjected to hydrogen reduction at an optimized temperature, successfully obtaining bulk porous HEAs (FeCoNiCu HEA) composed of single-phase solid solutions. Structural characterization revealed that the material possessed a rich pore structure and high specific surface area, exhibiting excellent catalytic performance in alkaline HER. Electrochemical tests showed that FeCoNiCu HEA required overpotentials of only 71 mV and 127 mV to achieve current densities of 10 mA·cm^−2^ and 50 mA·cm^−2^, respectively (Figure 9). Its catalytic activity was significantly superior to elemental, binary, and ternary alloy counterparts. After 1000 CV cycles or 50 h of constant current electrolysis, the material maintained stable catalytic performance, confirming its good electrochemical durability. Combined with DFT calculations, the mechanism of electronic interaction among Fe, Co, Ni, and Cu multi-components was revealed: The introduction of Fe optimized the d-band center position of Co/Ni active sites, while the presence of Cu reduced the water dissociation energy barrier by regulating surface charge distribution. The multi-element synergy significantly improved the adsorption/desorption kinetics of the H* intermediate. This study established a systematic method for preparing porous HEA catalysts via SCS combined with hydrogen reduction. By coupling component design with microstructure control, it provided a new strategy for developing efficient and stable HER catalysts, while the synthesis route has universal potential for expansion in multi-component catalytic material systems.

Lu et al. synthesized (Co_0.25_Ni_0.25_Mn_0.25_Zn_0.25_)Fe_2_O_4_ high-entropy oxide (HEO) electrodes via solution combustion synthesis and studied their OER catalytic performance [74]. They found that for non-activated HEO electrodes, the overpotential at a current density of 10 mA/cm^2^ was 276 mV. After a 100 h i-t test, the overpotential further decreased to 230 mV, a reduction of 46 mV. However, the CV activation process improved the OER catalytic performance. I-t testing revealed that the core of HEO particles maintained the spinel structure, but the flocculation around the core appeared as a combination of spinel and non-crystalline structures, likely due to selective element leaching and increased oxygen vacancies causing surface reconstruction.

Xie et al. prepared high-surface-area (CoMnFeCrNi)_3_O_4_ catalysts via solution combustion synthesis [75]. Subsequently, Pt/(CoMnFeCrNi)_3_O_4_ composite catalysts for C_3_H_8_ combustion were synthesized by atomic layer deposition. Catalytic testing in dry air (C_3_H_8_/O_2_/N_2_ = 1/10/89 vol%). Studies showed that Pt/HEO-EA achieved 90% propane conversion at 302 °C, while Pt/HEO-Gly and Pt/Al_2_O_3_ failed to achieve complete conversion even at 400 °C. HEO-EA exhibited excellent catalytic activity and water vapor resistance. The apparent activation energy (Ea) of Pt/HEO-EA was 80.9 kJ/mol, lower than Pt/HEO-Gly and Pt/Al_2_O_3_, indicating superior intrinsic catalytic activity. H_2_-TPR and O_2_-TPD tests indicated that Pt/HEO-EA had stronger activation and desorption capabilities for oxygen species at low temperatures. It was concluded that the high catalytic activity of Pt/HEO-EA was attributed to the easy activation of oxygen species at the Pt-HEO interface, promoting the decomposition of intermediates during propane combustion. This study demonstrates the potential of HEOs as supports for precious metal catalysts, providing a new strategy for developing efficient catalysts for low-temperature light hydrocarbon combustion.

### 3.3. Mechanical Alloying Method

Mechanical alloying (MA) for preparing HEA catalysts is an efficient solid-state non-equilibrium processing technique performed at room temperature. Its core principle is the use of mechanical forces from high-energy ball milling to achieve atomic-level mixing of multiple elements. Different metal powders undergo repeated cold welding, fracturing, and plastic deformation, ultimately breaking surface oxide layers and promoting atomic diffusion to form alloy powders with uniform nanostructures [76]. The specific process involves mixing equimolar or specific ratios of metal powders (e.g., Fe, Co, Ni, Cr, Mn) in a ball mill jar. Through violent collisions and friction of the grinding balls, powder particles experience cold welding to form layered composites. Subsequently, due to work hardening, they fracture, exposing fresh surfaces that weld again. This cycle repeats until atomic-level mixing is achieved, forming HEA powder with grain sizes refined to the nanometer scale. This method avoids the high energy consumption and element segregation problems of traditional melting methods, making it particularly suitable for multi-component systems with large melting point differences or thermodynamic immiscibility [77].

Pan et al. successfully prepared FeCoNiCrP high-entropy alloy powder via mechanical alloying [78]. Subsequently, the powder was loaded onto carbon fiber paper to fabricate an electrocatalyst for water electrolysis oxygen evolution. Studies found that the excellent performance of FeCoNiCrP was attributed to the high surface activity of the powder and its large specific surface area resulting from the high energy input during the MA process. Further electrochemical activation using cyclic voltammetry induced surface reconstruction of FeCoNiCrP. Electrochemical measurements revealed exceptional catalytic efficiency, achieving a current density of 10 mA cm^−2^ at merely 286 mV overpotential (η) with a Tafel slope of 27.6 mV dec^−1^. Cyclic voltammetry activation significantly expanded the electrochemical active surface area of the FeCoNiCrP alloy, thereby augmenting accessible active sites. Furthermore, this process induced in situ formation of oxyhydroxide floccules on particle surfaces, generating highly active metal (oxyhydr) oxide species that synergistically enhanced the OER performance.

**Figure 10:**
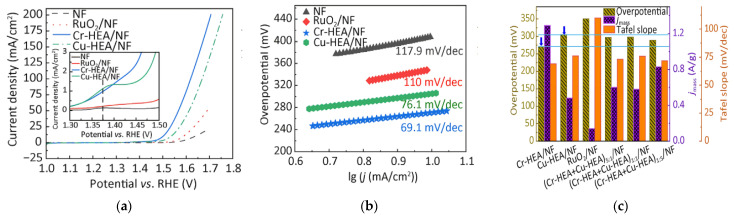

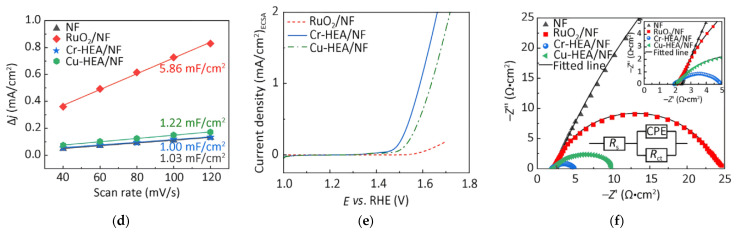
(**a**) Electrocatalytic performance of prepared Cr-HEA/NF, Cu-HEA/NF HEAs, NF, and RuO/NF electrocatalysts. OER polarization curves of tested electrocatalysts measured in 1 mol/L KOH solution, with IR compensation and local enlarged view; (**b**) Tafel plots; (**c**) Performance comparison at 10 mA/cm^2^ at 1.53 V (vs. RHE); (**d**) Capacitive current density vs. scan rate plots; (**e**) LSV from (**a**) normalized to ECSA; (**f**) Nyquist plot measured at overpotential 1.53 V (vs. RHE), equivalent circuit (lower part) and enlarged view (upper right). Reprinted with permission from Ref. [79]. Open access.

Wu et al. successfully prepared FeCoNiMoCr and FeCoNiMoCu high-entropy alloy powders using mechanical alloying and conducted in-depth studies [79]. The mechanically alloyed Cr-HEA and Cu-HEA powders possessed amorphous/BCC and FCC/BCC structures, respectively. Studies found that oxygen evolution reaction induced nanonization of the amorphous phase in the Cr-based HEA (Cr-HEA), simultaneously enhancing grain crystallinity. The Cr-HEA/NF electrode exhibited the best catalytic activity: The catalyst achieved benchmark current density (10 mA cm^−2^) at η = 271 mV, demonstrating a moderate Tafel slope (69.1 mV dec^−1^) with mass activity of 1.53 A mg^−1^ at 1.23 V vs. RHE. Its performance significantly surpassed that of the Cu-based HEA (Cu-HEA), commercial RuO_2_/NF, and bare NF electrodes. The catalyst operated stably for 48 h in 1 M KOH solution (10 mA/cm^2^), maintained a current density of 500 mA/cm^2^ under industrial-grade conditions (6 M KOH/85 °C) for 100 h, and showed only slight fluctuations during a 100 h constant current test (100 mA/cm^2^), confirming its exceptional long-term stability (Figure 10). Mechanistic studies indicated that synergistic effects among metal active sites enhanced catalytic activity by regulating the adsorption–desorption behavior of oxygen-containing intermediates. The introduction of chromium effectively modulated the electronic structure of the HEA, significantly reducing the energy barrier of the rate-determining step (ΔG_3_ values at 0 V and 1.23 V decreased), thereby lowering the overpotential. Furthermore, the multi-level nested heterogeneous interface formed between the amorphous phase and metastable nanocrystals, synergizing with the in situ generated multi-phase structure, collectively promoted the catalytic performance of Cr-HEA/NF.

### 3.4. Sol–Gel Method

The sol–gel method is a low-temperature synthesis technique for preparing inorganic or organic-inorganic hybrid materials through liquid-phase chemical reactions [80]. Its core steps involve the hydrolysis and condensation of precursors to form a sol, followed by gelation to form a three-dimensional network-structured gel. The target material is finally obtained through post-treatments like drying and sintering. Originating in the 1970s, this method is widely used in catalysts, ceramics, optical materials, etc., due to its mild reaction conditions (typically at room temperature to 100 °C), high product purity, and controllable microstructure (e.g., porosity, particle size, crystal phase).

**Figure 11:**
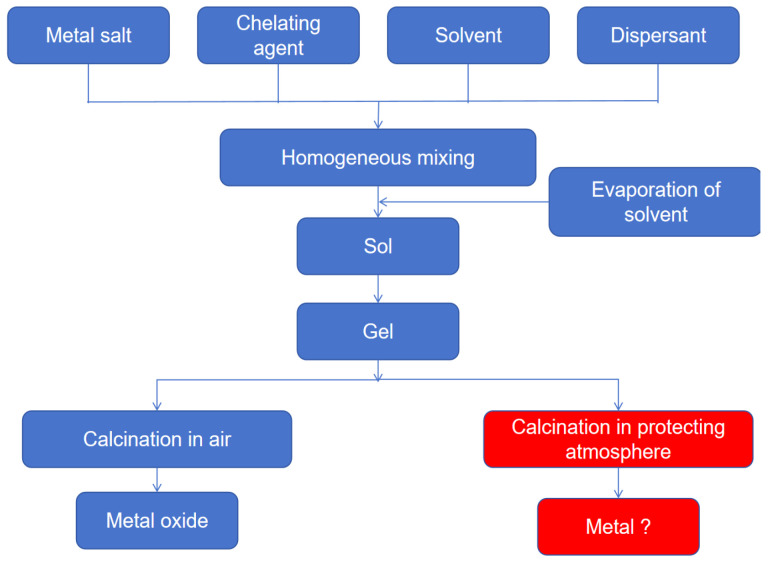
Implementation roadmap of the sol–gel method.

As a versatile liquid-phase synthetic approach, the sol–gel methodology enables precise control over compositional homogeneity and nanostructural evolution at near-ambient conditions, offering distinct benefits including: It enables the preparation of high-purity, composition-controllable multifunctional materials (e.g., nanoparticles, doped oxides, organic-inorganic hybrids) through molecular-level uniform mixing and low-temperature reaction conditions [81]. It also offers flexibility in shaping the product as films, fibers, or monoliths, being particularly suitable for coating complex substrates. Furthermore, its environmental friendliness is reflected in low energy consumption and minimal pollution. However, this method also has some disadvantages: Reliance on expensive metal alkoxides and toxic solvents increases cost and safety risks. The lengthy sol aging and drying processes can easily lead to material shrinkage and cracking. Residual micropores and organics require high-temperature calcination for removal, but excessive heat treatment may damage the material structure. The fine control of process conditions (e.g., pH, hydrolysis rate) further constrains large-scale production efficiency. Figure 11 below shows the implementation roadmap of the sol-gel method.

Tang et al. prepared MgMnFeCoNi B-site high-entropy perovskite cobaltites using the sol–gel method and comprehensively evaluated their OER electrocatalytic performance [82]. X-ray spectroscopic characterization indicated that changes in the charge states of multiple B-site cations and alternation in the electronic configuration of oxygen ions collectively promoted the enhancement of intrinsic OER activity. They also demonstrated that configurational entropy can serve as an effective tool to mitigate the formation of oxygen vacancies, greatly enhancing the OER on the electrocatalyst via the lattice oxygen oxidation mechanism pathway.

**Figure 12:**
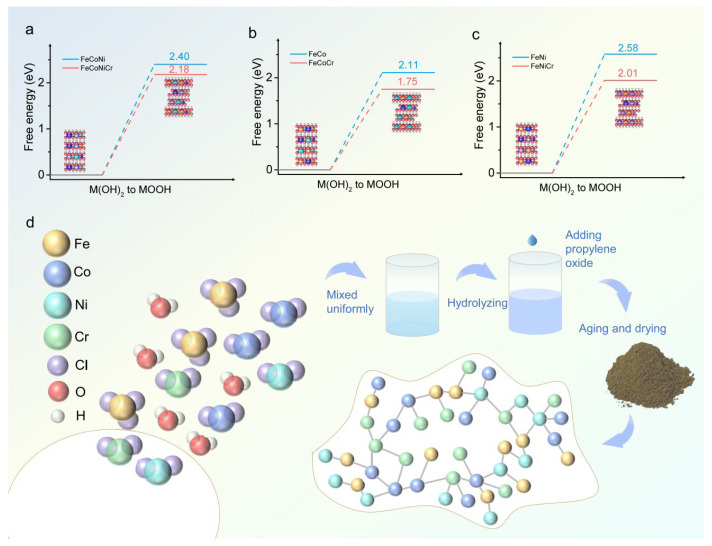
(**a**) Free energies of M(OH), to MOOH in FeCoNi and FeCoNiCr hydroxides. (**b**) Free energies of M(OH), to MOOH in FeCo and FeCoCr hydroxides. (**c**) Free energies of M(OH), to MOOH in FeNi and FeNiCr hydroxides. (**d**) Schematic illustration of FeCoNiCr hydroxide synthesis by aqueous sol–gel. Reprinted with permission from Ref. [83]. Open access.

Wang et al. also synthesized FeCoNiCr hydroxide via the sol–gel method. Studies revealed that FeCoNiCr hydroxide exhibited a very low overpotential of 224 mV in alkaline media, 52 mV lower than Cr-free FeCoNi hydroxide, and demonstrated outstanding comprehensive performance in the oxygen evolution reaction (OER) [83]. Experimental tests showed its Tafel slope was only 42.7 mV dec^−1^, significantly lower than typical metal oxide catalysts (e.g., 74.9 mV dec^−1^ for RuO_2_). This excellent kinetic characteristic stemmed from the catalyst’s optimized regulation of the adsorption energy of OER intermediates, bringing the reaction path closer to the theoretical optimum. In terms of stability, the material maintained continuous operation for over 150 h at 10 mA cm^−2^, and its polarization curve remained similar to the initial state after 500 cycles, with an overpotential shift of less than 5%, meeting industrial-grade catalyst requirements for durability (Figure 12).

When applied in a zinc–air battery system, the catalyst exhibited a discharge/charge voltage difference of 0.70 V and showed no voltage drop or catalyst structure collapse during 160 h of continuous cycling. This low polarization characteristic is closely related to its bifunctional catalytic activity, with its voltage difference metric significantly superior to the traditional Pt/C+Ir/C system (initial difference 0.8 V, failure after 400 cycles). It is important to note that the stability of battery performance is strongly correlated with the intrinsic stability of the catalyst. X-ray absorption spectroscopy (XAS) verified the preservation of atomic-scale structural integrity at metal coordination centers throughout electrochemical cycling, demonstrating unaltered local environments compared to the pristine catalyst.

The following Table 3 presents the composition, application scenarios, and preparation methods of several high-entropy alloys. Table 4 provides a brief assessment of these preparation methods.

## 4. Applications of High-Entropy Alloys in Catalysis

With the urgent demand for efficient electrocatalytic materials driven by the energy transition, high-entropy alloys have emerged as a new paradigm to overcome the performance bottlenecks of traditional catalysts, leveraging their multi-element synergistic effects and structural tunability. Traditional precious metal catalysts are difficult to meet large-scale application demands due to high cost, resource scarcity, and limitations of single active sites. In contrast, HEAs, through lattice distortion and the “cocktail effect” among multi-element atoms, exhibit high activity, strong stability, and cost advantages in reactions such as hydrogen evolution, oxygen evolution, and oxygen reduction. Their high-entropy characteristics suppress element segregation and optimize electronic structure. Combined with nanoscale design exposing abundant active sites, they provide novel approaches for regulating the kinetics and thermodynamics of electrocatalytic reactions.

### 4.1. Hydrogen Evolution Reaction (HER)

The hydrogen evolution reaction (HER) is a key cathodic step in water electrolysis. Its core is the reduction of hydrogen ions or water molecules to hydrogen gas via electron transfer. The reaction follows the Volmer–Heyrovsky or Volmer–Tafel mechanism, where the Volmer step involves hydrogen atom adsorption, and the Heyrovsky or Tafel steps generate H_2_ via electrochemical or chemical desorption, respectively [89].

According to recent research progress, HEA catalysts significantly enhance HER efficiency while reducing dependence on precious metals through synergistic effects of multi-metal components and optimized structural design. For example, Jia et al. successfully synthesized PtRuCoNiCu HEA nanoparticles via low-temperature thermal reduction [85]. Studies found that in PtRuCoNiCu HEA, Pt and Ru served as primary active sites directly involved in the reaction, while Co, Ni, and Cu promoted the adsorption/desorption process of the H* intermediate by modulating the electronic structure, accelerating H_2_ generation and release. It exhibited tremendous HER activity and stability in acidic media, promoting synergistic charge/mass transfer kinetics and improving metal-support interactions, leading to enhanced HER activity and stability while reducing precious metal usage.

Zhao et al. synthesized high-entropy alloy PtPdRhRuCu nanoparticles using solution combustion synthesis [84]. They found that the PtPdRhRuCu/C catalyst achieved a mass activity of 3.0 A mg^−1^ Pt in 1 mol/L KOH electrolyte, 7.9 times higher than commercial Pt/C (0.38 A mg^−1^Pt). Compared to conventional multi-component alloys, this HEA exhibited superior catalytic activity and stability in HER. The enhanced catalytic performance stems from cooperative interactions among multimetallic active sites. Operando synchrotron X-ray absorption spectroscopy tracking of Cu/Pt oxidation states and coordination geometries confirmed the preservation of atomic-scale disorder in PtPdRhRuCu/C throughout alkaline electrolysis, with no detectable phase segregation or elemental redistribution. Density functional theory (DFT) calculations further elucidated its catalytic mechanism: Ru sites optimized the H_2_O adsorption configuration, lowering the water dissociation energy barrier (0.62 eV); Pt sites acted as proton capture centers accelerating H* intermediate desorption; while Cu-induced lattice contraction weakened the bonding strength of adsorbed species. The synergistic action of these three significantly optimized the thermodynamic pathways of the Volmer step (water dissociation) and Heyrovsky step (H_2_ generation).

### 4.2. Oxygen Evolution Reaction (OER)

The oxygen evolution reaction (OER), functioning as the anodic counterpart in water electrolysis cells, proceeds through a quaternary proton-coupled electron transfer pathway with concomitant O-O bond formation. This multi-step process imposes substantial kinetic barriers, necessitating significant overpotentials to drive practical reaction rates. The reaction mechanisms are categorized into adsorbate evolution mechanism (AEM) and lattice oxidation mechanism (LOM): AEM completes via stepwise deprotonation of OH→O→OOH* intermediates, while LOM relies on the oxidative coupling of lattice oxygen.

Ma et al. treated a high-entropy alloy (HEA) with HF to hydroxylate it (HF-HEA) and performed in situ electrochemical activation, successfully preparing a novel highly efficient CoCrFeNiAl HEA-supported Co, Fe, Ni-(O)OH electrocatalyst [86]. This catalyst demonstrated remarkable electrocatalytic efficacy for the oxygen evolution reaction, achieving the benchmark current density of 10 mA cm^−2^ at an overpotential η = 240 mV, with a Tafel slope of 52.7 mV dec^−1^. Studies showed that Cr and Al elements played key promoting roles in the HEA/Co, Fe, Ni-(O)OH system, not only enhancing its electrochemical activity but also further boosting catalytic performance by increasing the Ni^3+^/Ni^2+^ molar ratio on the Ni(OH)_x_/NiOOH surface. Furthermore, during electrochemical activation, the leaching of Cr^3+^ and Al^3+^ significantly increased the electrochemical active surface area (ECSA) of the HF-HEA, thereby improving overall catalytic efficiency. This research provides new insights for the application of HEAs in electrocatalysis and is expected to advance their development in high-efficiency electrocatalysts.

Zhou et al. addressed the issues of expensive and inefficient electrodes and the exceptionally low efficiency of hydrogen production in alkaline water electrolysis (AWE) by using AlCoCrFeNi high-entropy alloy as a high-efficiency electrode [90]. They found that after anodic oxidation treatment of the HEA in 1 mol L^−1^ HCl, its surface consisted of metals, oxides, and oxyhydroxides, forming some pore structures. Subsequently, in 1 mol L^−1^ KOH solution, the anodized HEA exhibited excellent hydrogen evolution reaction (HER) and oxygen evolution reaction (OER) performance. Its HER overpotential was only 84.5 mV at 500 mA cm^−2^, and OER overpotential was 88.0 mV at −500 mA cm^−2^. In a full water-splitting cell, this electrocatalyst achieved a current density of 500 mA cm^−2^ at an overpotential of 3.00 V and maintained good stability even after prolonged operation (over 100 h). In situ Raman spectroscopy analysis further revealed the critical role of multi-metal elements in HER, while multi-metal oxyhydroxides dominated the OER. Additionally, the bulk HEA structure formed by anodization shows great potential in industrial-scale AWE systems, promising to be an efficient and stable electrode material.

### 4.3. Oxygen Reduction Reaction (ORR)

The oxygen reduction reaction (ORR) is the core cathodic process in fuel cells, where oxygen is reduced to water or hydrogen peroxide via a four-electron or two-electron pathway. The four-electron pathway is highly valued due to its high energy efficiency but requires overcoming the high activation energy for O_2_ dissociation. The reaction pathway selectivity is predominantly governed by the catalyst’s oxygen adsorption energetics and kinetic facility for O-O bond scission.

Qiu et al. used a simple alloying-dealloying process to uniformly mix five different metal elements in variable combinations and compositions, forming a nanoscale body-centered cubic (BCC) structure phase [87]. The prepared HEAs with low Pt content (approx. 20–30 at.%) exhibited a bimodal porous structure and ultra-fine multi-component alloy nanochains. By studying and screening the composition effect, a highly ORR-active np-AlCuNiPtMn catalyst was discovered, exhibiting a half-wave potential of 0.945 V in acidic media and a mass activity approximately 16 times higher than Pt/C. This np-HEA offers a vast design space, providing a new pathway for discovering high-quality catalysts for different chemical reactions [88].

Further research showed that the dealloying strategy can efficiently prepare HEA materials with porous structures, significantly increasing their specific surface area and exposing more active sites, thereby enhancing electrocatalytic performance. For instance, Lee et al. successfully synthesized various nanoporous HEAs, including AlNiCuPtPdAu, by combining melt spinning and dealloying [91]. They found that the AlCuNiPtMn HEA outperformed Pt/C catalyst in ORR catalytic activity and electrochemical cycling stability. Moreover, the np-HEA exhibited excellent durability in acidic solution, maintaining 92.5% of its initial activity even after 100 electrochemical cycles. The following Table 5 lists the performance comparison of high-entropy alloy catalysts in three reactions. 

## 5. Conclusions

High-entropy alloy catalysts (HEAs), leveraging their unique high-entropy effect, lattice distortion effect, sluggish diffusion effect, and “cocktail effect,” have broken through the design limitations of traditional alloys. As multi-principal element near-equimolar solid solutions, they exhibit significant comprehensive performance advantages in the field of electrocatalysis. Their flexible tunability in composition and structure provides broad scope for optimizing electronic states, exposing high-density active sites, and regulating the adsorption behavior of key intermediates. The development of various preparation methods (e.g., chemical co-reduction, solution combustion, mechanical alloying, sol–gel), combined with theoretical calculations and data-driven strategies, has further propelled material design and performance enhancement. In core electrocatalytic reactions such as hydrogen evolution, oxygen evolution, and oxygen reduction, HEAs demonstrate activity and stability surpassing traditional catalysts. Their performance improvement stems from the optimization of reaction pathways and kinetics through multi-element synergy.

Future research needs to deeply reveal the dynamic reaction mechanisms at multi-component surface active sites and develop cross-scale theoretical calculations and machine learning-assisted rational design methods to accelerate the development of high-performance non-precious metal HEA systems. Simultaneously, bottlenecks in large-scale preparation concerning compositional uniformity, structural stability, and cost control need to be overcome. Nanostructure design and surface/interface engineering should be optimized, and their application potential in complex practical systems (e.g., industrial-grade electrolysis, fuel cells) needs to be expanded. Establishing unified and rigorous performance evaluation standards is key to realizing the transition of HEA catalysts from fundamental research to industrial application. With deepening mechanistic understanding and innovation in preparation technologies, HEA catalysts are expected to provide breakthrough solutions for clean energy conversion and storage technologies.

Future work must address: (i) to address kinetic energy bottlenecks at multi-elemental active sites by in situ spectroscopy; (ii) the discovery of low-cost, non-precious metal-based mixed-element alloy systems through machine learning guidance; (iii) challenges in industrial-scale synthesis (e.g., composition uniformity in the sol–gel route); (iv) Standardized test protocols for industrial benchmarking.

## Figures and Tables

**Table 1:** Application scenarios of electrocatalysts.

Reaction Type	Catalyst Examples	Target Function	Reference
Water Electrolysis for H_2_ Production	Pt, MoS_2_, Ni-Mo Alloy	Efficient H_2_ Production, Lower Overpotential	[10]
CO_2_ Reduction	Cu Nanowires, Ag Nanoparticles	Selective Generation of CO, CH_4_, or C_2_^+^ Products	
Oxygen Reduction Reaction	Pt-Co Alloy, Fe-N-C Materials	Enhance Fuel Cell Efficiency, Reduce Precious Metal Usage	[11]
Oxygen Evolution Reaction	IrO_2_, NiFe-LDH	Accelerate Water Splitting, Extend Electrolyzer Lifespan	
Nitrogen Reduction for Ammonia Synthesis	Ru-Based Materials, Bi Nanosheets	Activate N_2_ to Generate NH_3_ at Ambient Temperature and Pressure	[12]

**Table 2:** Comparison of Common Catalysts.

Catalyst Type	Advantages	Disadvantages	Typical Applications
Precious Metal-Based	Ultra-high Activity, Good Stability High	Cost, Resource Scarcity	Fuel Cells, Water Electrolysis
Transition Metal-Based	Low Cost, High Tunability	Lower Activity, Poor Stability	Alkaline Water Electrolysis, CO_2_RR
MOFs/COFs	High Specific Surface Area, Designable Structure	Poor Conductivity, Insufficient Stability	CO_2_RR, OER
Carbon-Based Materials	Good Conductivity, Environmentally Friendly	Dependence on Doping, Structure Prone to Collapse	ORR, Metal–Air Batteries
Single-Atom Catalysts	High Atom Utilization, Excellent Selectivity	Complex Synthesis, Easy Deactivation	High-Selectivity Reactions (e.g., NRR)
Non-Metallic Catalysts	Metal-Free, Corrosion Resistant	Low Activity, Narrow Applicability	Photo/Electrocatalytic Water Splitting

**Table 3:** HEA compositions and applications.

Application	Composition	Preparation Method	Reference
HER	NiCoFePtRh	Chemical Co-reduction	[45]
Methanol/Formic Acid Electrooxidation	PtCuNiCoMn	Chemical Co-reduction	[62]
HER	FeCoNiCu	Solution Combustion Synthesis	[65]
OER	FeCoNiCrP	Mechanical Alloying	[78]
HER	CoFeCuMnZn	Solution Combustion Synthesis	[73]
OER	FeCoNiMoCr	Mechanical Alloying	[79]
OER	MgMnFeCoNi	Sol–Gel	[82]
OER	FeCoNiCr	Sol–Gel	[83]
HER	PtPdRhRuCu	Solution Combustion Synthesis	[84]
HER	PtRuCoNiCu	Low-Temperature Thermal Reduction	[85]
OER	CoCrFeNiAl	Hydroxylation via HF treatment	[86]
ORR	AlCuNiPtMn	Dealloying	[87]
ORR	AlNiCuPtPdAu	Dealloying	[88]
CO2RR	AuAgPtPdCu	Low-Temperature Method	[57]
NRR, OER	Ni, Fe, Co, Mn, V	Solution Combustion Synthesis	[31]
HOR	PtRuNiCoFeMo	Solution Combustion Synthesis	[47]
Propane Combustion	CoMnFeCrNi	Solution Combustion Synthesis	[75]

**Table 4:** Comparative assessment of HEA synthesis methods.

Method	Advantages	Limitations	Suitability for Electrocatalysis
Chemical Co-reduction	Uniform nanoparticles (1–3 nm); Precise composition control	Toxic reagents; Scalability challenges	High (e.g., HER/ORR nanocatalysts)
Solution Combustion	Rapid; Porous structures; Scalable	Residual carbon/impurities; Composition drift	Moderate (e.g., OER oxides)
Mechanical Alloying	Solvent-free; Bulk production	Elemental segregation (Figure 10c); Contamination risk	Low (requires post-annealing for homogeneity)
Sol–Gel	Homogeneous doping; Thin-film compatibility	Long processing; High cost	High (e.g., perovskite OER catalysts)

**Table 5:** Performance comparison of HEA catalysts in key reactions.

Reaction	HEA Composition	Overpotential (η)	Tafel Slope	Stability	Conditions	Reference
HER	NiCoFePtRh/C	28 mV @ 10 mA cm^−2^	30.1 s^−1^ TOF	>99% (10k cycles)	0.5 M H_2_SO_4_, 25 °C	[45]
OER	FeCoNiCrP	286 mV @ 10 mA cm^−2^	27.6 mV dec^−1^	>24 h @ 100 mA cm^−2^	1 M KOH, 25 °C	[78]
ORR	np-AlCuNiPtMn	E_1_/_2_ = 0.945 V	Unclear	92.5% (100 cycles)	0.1 M HClO_4_, 25 °C	[89]
HER	FeCoNiCu	71 mV @ 10 mA cm^−2^	45 mV dec^−1^	>50 h @ 50 mA cm^−2^	1 M KOH, 25 °C	[65]

## Data Availability

No new data were created or analyzed in this study. Data sharing is not applicable to this article.
